# High-resolution label-free mapping of murine kidney vasculature by raster-scanning optoacoustic mesoscopy: an ex vivo study

**DOI:** 10.1186/s40348-022-00144-0

**Published:** 2022-07-04

**Authors:** Colin A. Goebel, Emma Brown, Fabian B. Fahlbusch, Alexandra L. Wagner, Adrian Buehler, Thomas Raupach, Martin Hohmann, Moritz Späth, Neal Burton, Joachim Woelfle, Michael Schmidt, Andrea Hartner, Adrian P. Regensburger, Ferdinand Knieling

**Affiliations:** 1 Department of Pediatrics and Adolescent Medicine, University Hospital Erlangen, Friedrich-Alexander-Universität (FAU) Erlangen-Nürnberg, Erlangen, Germany; 2 Department of Physics, University of Cambridge, Cambridge, UK; 3 Cancer Research UK Cambridge Institute, University of Cambridge, Cambridge, UK; 4 Washington University School of Medicine, St. Louis, USA; 5 Institute of Photonic Technologies, Friedrich-Alexander-Universität Erlangen-Nürnberg, Erlangen, Germany; 6 Erlangen Graduate School in Advanced Optical Technologies, 91052 Erlangen, Germany; 7 iThera Medical GmbH, München, Germany

**Keywords:** RSOM, msRSOM, Raster scanning optoacoustic mesoscopy, Optoacoustic, Photoacoustic, Ex vivo kidney imaging, Chronic kidney disease

## Abstract

**Background:**

Chronic kidney disease (CKD) is a global burden affecting both children and adults. Novel imaging modalities hold great promise to visualize and quantify structural, functional, and molecular organ damage. The aim of the study was to visualize and quantify murine renal vasculature using label-free raster scanning optoacoustic mesoscopy (RSOM) in explanted organs from mice with renal injury.

**Material and methods:**

For the experiments, freshly bisected kidneys of alpha 8 integrin knock-out (KO) and wildtype mice (WT) were used. A total of *n*=7 female (*n*=4 KO, *n*=3 WT) and *n*=6 male animals (*n*=2 KO, *n*=4 WT) aged 6 weeks were examined with RSOM optoacoustic imaging systems (RSOM Explorer P50 at SWL 532nm and/or ms-P50 imaging system at 532 nm, 555 nm, 579 nm, and 606 nm). Images were reconstructed using a dedicated software, analyzed for size and vascular area and compared to standard histologic sections.

**Results:**

RSOM enabled mapping of murine kidney size and vascular area, revealing differences between kidney sizes of male (m) and female (f) mice (merged frequencies (MF) f vs. m: 52.42±6.24 mm^2^ vs. 69.18±15.96 mm^2^, *p*=0.0156) and absolute vascular area (MF f vs. m: 35.67±4.22 mm^2^ vs. 49.07±13.48 mm^2^, *p*=0.0036). Without respect to sex, the absolute kidney area was found to be smaller in knock-out (KO) than in wildtype (WT) mice (WT vs. KO: MF: *p*=0.0255) and showed a similar trend for the relative vessel area (WT vs. KO: MF *p*=0.0031). Also the absolute vessel areas of KO compared to WT were found significantly different (MF *p*=0.0089). A significant decrease in absolute vessel area was found in KO compared to WT male mice (MF WT vs. KO: 54.37±9.35 mm^2^ vs. 34.93±13.82 mm^2^, *p*=0.0232). In addition, multispectral RSOM allowed visualization of oxygenated and deoxygenated parenchymal regions by spectral unmixing.

**Conclusion:**

This study demonstrates the capability of RSOM for label-free visualization of differences in vascular morphology in ex vivo murine renal tissue at high resolution. Due to its scalability optoacoustic imaging provides an emerging modality with potential for further preclinical and clinical imaging applications.

**Supplementary Information:**

The online version contains supplementary material available at 10.1186/s40348-022-00144-0.

## Introduction

Chronic kidney disease (CKD) is a major cause of morbidity and mortality, which affects almost 700 million people representing 9.1% of the global population [1]. From that, approximately 15–74.7 million cases occur in children and adolescents [2–5]. In comparison to adults, wherein arterial hypertension or diabetes mellitus are the main etiologies of CKD [1, 6, 7], most pediatric cases originate from congenital disorders [3, 8]. At present, the only available option to obtain specific information about the extent of altered kidney tissue is derived from kidney biopsy and histological analysis. This requires invasive removal of a small tissue sample, while at the same time, it only represents a small proportion of the organ as a whole [9]. In clinical practice, the degree of renal dysfunction is determined by estimating glomerular filtration rate (GFR) from blood samples [10, 11]. In contrast to laboratory measurements, imaging enables direct visualization and quantification of kidney tissue remodeling, inflammation, and fibrosis [10, 12–14]. However, various imaging techniques are gaining momentum to objectively assess kidney structure and function [11, 14, 15]. Advanced imaging techniques, such as magnetic resonance imaging (MRI), are expensive and challenging in the youngest patients by the need of sedation and the associated risks [16]. Therefore, there is an unmet clinical need for novel imaging biomarkers in pediatric medicine to diagnose and monitor structural, functional, and molecular changes during CKD progression. In this regard, raster-scanning optoacoustic mesoscopy (RSOM) offers the opportunity for non-invasive, agent-free tissue imaging by the photo-/optoacoustic effect [17, 18]. Using a pulsed laser light source to excite tissue chromophores such as hemoglobin, RSOM detects acoustic pressure waves generated from thermoelastic expansion of these molecules [19, 20]. Imaging with RSOM has currently gained interest for imaging human skin diseases [21]. The purpose of this pilot study was to visualize and quantify changes in renal vascular morphology by RSOM in healthy mice and mice with renal injury [22].

## Methods

### Animal model

According to standard conditions, all mice were bred and maintained with free access to tap water and standard rodent chow in a room maintained at 22±2 °C exposed to a 12-h dark/light cycle. For imaging experiments, alpha 8 integrin knock-out (129/Sv itga8^-/-^, KO) and wildtype (itga8^+/+^, WT) litters were used at an age of 6 weeks. A total of *n*=7 female (*n*=4 KO, *n*=3 WT) and *n*=6 male animals (*n*=2 KO, *n*=4 WT) were included.

### Tissue preparation and histology

For experiments, the mice were sacrificed under isoflurane anesthesia. The left and right kidneys, if available [23], were harvested and bisected. Immediately thereafter, the kidneys were stored in PBS (phosphate-buffered saline; PAN-Biotech, Aidenbach, Germany) for 10 s. After imaging was completed, one half of the kidney was transferred to fixation medium (Methyl-Carnoy fixative) for histology. For histologic comparisons, tissue sections were cut (3μm) and stained with a laboratory standard protocol for periodic acid-Schiff’s (PAS) reagent [24]. For quantification of the renal vasculature, immunohistochemical staining of smooth muscle actin (SM-Actin) 1A4 (DAKO, Hamburg, Germany) for arterioles and endomucin (Venous Endomucin (V.7C7) 1:50, Santa Cruz Biotechnology Inc., USA) for capillaries was used. Antibodies were applied to deparaffinized tissue sections. Vascular cross-sections were counted in 5 medium-powered cortical views (SM-Actin, 1:200) and 10 high-powered cortical views (Endomucin, 1:400) per renal cross-section.

### Imaging studies

#### Raster-scanning optoacoustic mesoscopy (RSOM)

For optoacoustic ex vivo kidney imaging, an RSOM Explorer P50 imaging system (iThera Medical GmbH, München, Germany) was used [25, 26]. Briefly, the bisected kidney was placed in the middle of a Petri dish filled with agarose 2% gel (Biozym LE Agarose, Biozym Scientific GmbH, Oldendorf, Germany) in order to reduce reflections and imaging artifacts. The portion of the kidney that was cut was placed flatly against the membrane of the detector head to enable artifact-free imaging, the sample was covered with centrifuged bubble-free ultrasound gel (medimex ultrasound gel; medimex GmbH, Limburg, Hessen, Germany), and the scan head was coupled by an interchangeable water-filled (2 ml) interface [27]. Each scan was performed in a light-tight imaging chamber containing a heated sample bed. Through a custom-made two-arm fiber bundle, the sample was illuminated at 532 nm [19, 20]. Detection of ultrasound signals (11–99MHz) from the sample was achieved by a customized spherically focused LiNbO3 detector. Using a motorized stage, scans were acquired with a field of view of 12 x 12 x 3 mm^3^ at 10 μm axial and 40 μm lateral resolution using a system laser energy of 95%. To verify the correct positioning and detector-to-sample-distance, a pre-scan was performed before every scan, detailed technical data is in Supplementary Table 1 [19, 26, 28, 29].

#### Multispectral RSOM (msRSOM)

For multispectral imaging, an RSOM Explorer ms-P50 imaging system (iThera Medical GmbH, Munich, Germany) was used. In contrast to the P50 system with 532 nm, the ms-P50 uses additional wavelengths at 555, 579, and 606 nm. The scanning head only consists of a single custom-made fiber from which the laser light is delivered to the sample. A similar spherically focused LiNbO3 through-hole detector was used for the detection, as described in the detailed technical data in Supplementary Table 2. The reconstructed images were spectrally unmixed by linear regression for HbR and HbO_2_ using all four available wavelengths [30, 31].

### Data analysis and image visualization

**Fig. 1:**
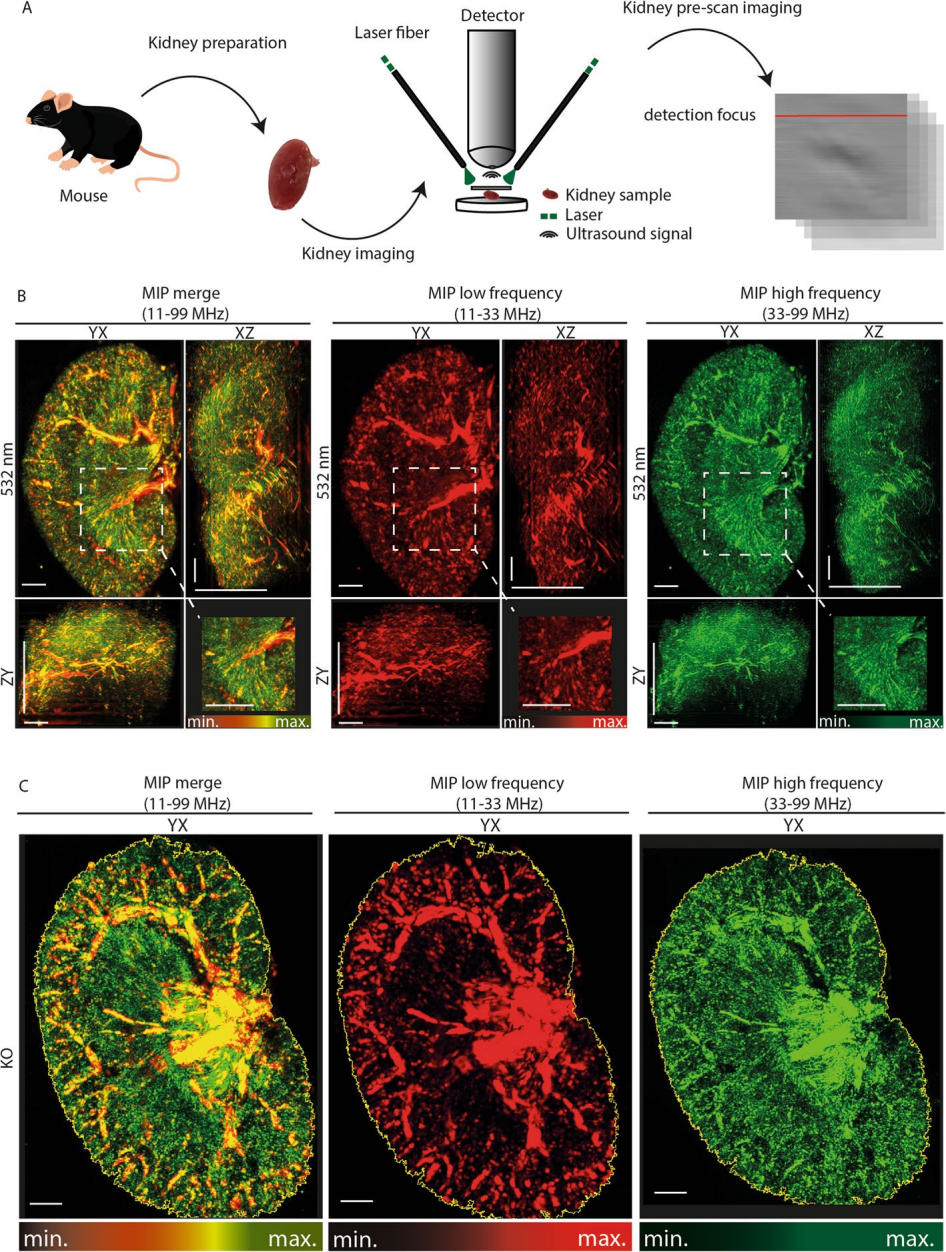
RSOM imaging work flow and exemplary images of renal vasculature. **A** Schematic cartoon of the imaging workflow. After cervical dislocation, kidneys were prepared and freshly imaged with the RSOM system. The excised kidney is covered with ultrasound gel and placed under a water bed shielded by a plastic foil. The tissue is illuminated with two 532nm lasers and the generated ultrasound waves are captured by the detector. A pre-scan is conducted before every full scan. Optoacoustic signals are than reconstructed by a dedicated software visualizing mainly haemoglobin molecules and the respective structures filled with it. **B** Exemplary reconstructed cross-sectional RSOM maximum intensity projections (MIP) of a heterozygotes (HET) mouse kidney are displayed. Merged frequencies (red-green) combine the information from low frequencies (bigger vessels = red) and higher frequencies (smaller vessels = green) allowing visualization of renal vasculature. All three imaging planes (YX, XZ, and ZY) are displayed with a 2-fold magnification of a central kidney section (Rectangle 2x2mm) with a large and many small vessels. Scale bars indicate 1mm. **C** Exemplary reconstructed cross-sectional maximum intensity projections (MIP) of a knockout (KO) mouse kidney are displayed. A region of interest (outlined in yellow) was automatically drawn around each kidney. The included area was used for further analyses. HET = heterozygotes (HET), KO = knockout (KO), MIP = maximum intensity projection, ROI = region of interest, MF = merge frequency, LF = low frequency, HF = high frequency, min.= minimum, max.= maximum

For image analyses, dedicated software (rLabs, version 1.19.04 for RSOM data, msrLabs, version 1.21.04 for msRSOM data, iThera Medical GmbH, Munich, Germany) was used to separate every RSOM imaging stack into two frequency bands: lower frequency (11–33 MHz (coded red; LF)) and higher frequency (33–99 MHz (coded green; HF)) [25, 26, 32]. As described before [33], this bandwidth separation was done for all images to allow for visualization of larger (LF) and smaller (HF) vessels. For each volume, three different recording planes (YX ~12 x 12 mm, XZ ~ 12 x 2.8 mm, YZ ~ 12 x 2.8 mm - orientation) were visualized as maximum intensity projections (MIP). Frequency division into LF and HF sub-bands was used to distinguish between larger (red) and smaller (green) vessels, respectively. Using a commercially available unmixing algorithm for oxygenated and deoxygenated hemoglobin (HbO_2_/HbR) [30, 31], differently oxygenated parenchymal regions could be visualized. This data set (HbO_2_ coded in red and HbR coded in blue) was merged, and the scaling was manually adjusted for better visualization. The 3D volume of the kidney was obtained from the stacked images using Amira (Version 6.4, Thermo Fisher Scientific, Zuse Institute Berlin, Germany). For further analyses, the exported RSOM images were converted to grayscale images via FIJI (V.1.8.0_172/1.52p. Wayne Rasband, National Institutes of Health, Maryland, USA) [34]. Then, a MIP was created with approx. 480 slices per stack. For quantification, individual regions of interest (ROI) were automatically drawn after the pixel area to be measured was manually defined by the user with a greyscale threshold setting (scaling 0 to 255). The content of the ROI was used for analysis (Fig. 1). The following parameters were analyzed: absolute kidney area, absolute vessel area, and relative vessel area (absolute vessel area/absolute kidney area). Within the ROI, the area marked by the grayscale threshold could be used for the measurement of relative and absolute vessel area.

### Statistical analysis

The mean and standard deviation (± SD) of all kidney parameters were determined. Before analysis, the data were tested for normal distribution using the Shapiro-Wilk test. Unpaired analysis was performed to compare the means of the two groups. In case of normal data distribution, an unpaired *t* test was used, and for non-normal distribution, a Mann-Whitney test was applied. The results were considered statistically significant at a *p*-value <0.05.

The raw data was recorded in Excel (Microsoft Office Professional Plus 2016, Redmond, Washington, USA). Statistical analyses were performed with GraphPad Prism (Version 9.3.1, GraphPad Software, Inc., La Jolla, California, USA).

## Results

### Visualization of kidney size and vasculature

**Fig. 2:**
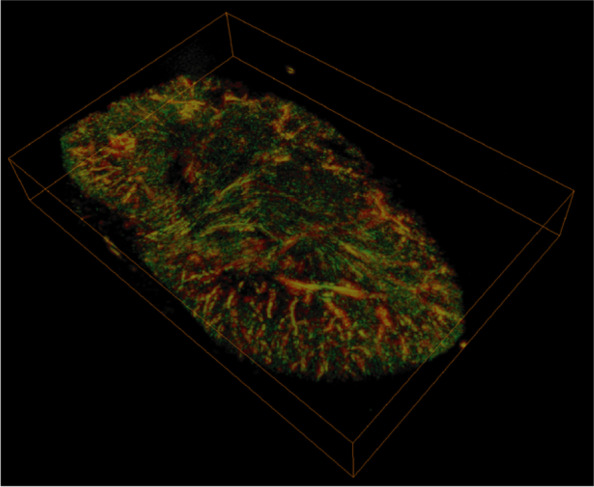
3D Visualization of an entire organ. 3D visualization of an entire kidney. Merged frequencies (red=lower frequencies, bigger vessels; green=higher frequencies, smaller vessels) provide information about the position of the partially overlapping big and small vessels and the signal strength of the different emitted signals by the amount of blood from different vessel sizes

A total of *n*=23 bisected kidneys were imaged with label-free RSOM (Fig. 1A). After image reconstruction, OAI signals at 532nm, reflecting mainly hemoglobin in the vascular system, were used for further analyses. Depending on the evaluated ultrasound frequency bands, bigger (low frequencies) or smaller (high frequencies) vessels can be visualized. By merging both frequency bands the entire vasculature can be illustrated (Fig. 1B). The clear borders of OAI signals were used to determine the region of interest for further analyses (Fig. 1C). An illustration of the 3-dimensional volume of the kidney provides additional information about the distribution of the vessels (Fig. 2). All kidney parameters were evaluated for low frequency (LF), high frequency (HF), and merged frequency (MF) bands. In the following, results are presented for MF. First, the total kidney area, relative vessel area, and absolute vessel area were analyzed, showing no difference between the left (*n*=10) and right (n=sidesside (all *p* >0.05) (Supplementary Figure 1A).

When comparing absolute kidney areas between sexes, independent from genotype, male kidneys were larger than female kidneys (*n*=12 female vs. *n*=11 male, MF: 52.42±6.24 mm^2^ vs. 69.18±15.96 mm^2^, *p*=0.0156). A similar trend was seen for the absolute vessel area (female vs. male MF: 35.67±4.22 mm^2^ vs. 49.07±13.48 mm^2^, *p*=0.0036). The relative vessel area did not differ significantly between males and females (all p>0.05) (Supplementary Figure 1B and Supplementary Table 3). In WT mice only, the absolute kidney area and absolute vessel area in male kidneys were found to be larger than in female kidneys (*n*= 5 female kidneys vs. *n*=8 male kidneys, MF, kidney area: 51.06±3.64 mm^2^ vs. 75.51±9.82 mm^2^, *p*=0.0003; kidney vessel area 36.39±3.18 mm^2^ vs. 54.37±9.35 mm^2^, *p*=0.0018) (Supplementary Figure 2).

### Kidney size and vascularization compared between different genotypes

Next, the ability of RSOM to assess disease-related changes was evaluated. Without respect to sex, the absolute kidney area was found to be smaller in KO than in WT mice (*n*=13 WT vs. *n*=10 KO: MF: 66.11±14.63 mm^2^ vs. 53.06±10.84 mm^2^ *p*=0.0255) and also showed a similar trend for the relative vessel area (WT vs. KO: MF 71.49±3.92 vs. 66.04±3.84 mm^2^ *p*=0.0031). Also the absolute vessel areas of KO compared to WT were found significantly different (WT vs. KO: MF 47.45±11.72 vs. 35.09±7.70 mm^2^ *p*=0.0089) (Supplementary Figure 3 and Supplementary Table 4).

### Genotype effect on kidney size and vascularization for different sexes

**Fig. 3:**
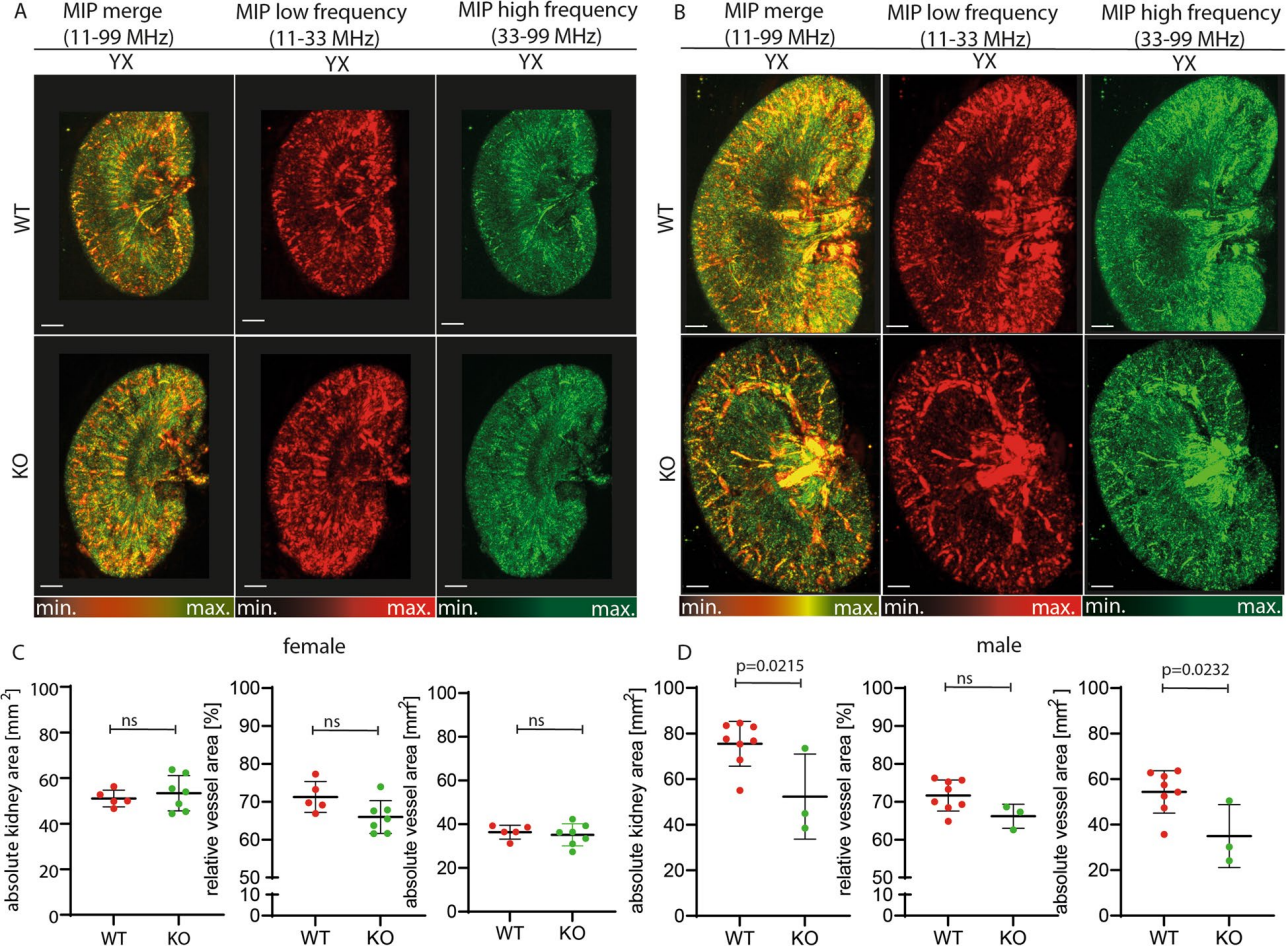
Ex vivo RSOM images of healthy compared to KO mice. **A**, **B** Exemplary MIP images of female (**A**) and male (**B**) kidneys are shown. Top row WT genotype, bottom row KO genotype. Merged frequencies (big and small vessels, red-green), low frequencies (bigger vessels = red), and higher frequencies (smaller vessels = green) are shown. Scale Bars 1mm. **C**, **D** WT and KO female (**C**) and male (**D**) kidneys were compared for absolute kidney area in mm^2^, relative vessel area, and absolute vessel area in mm^2^ derived from merged images. Bars and whiskers mark mean±SD. A p value <0.05 indicates statistical significance. ns=not significant, MIP=maximum intensity projection. LF=low frequencies, HF=high frequencies, MF=merged frequencies, WT=wildtype genotype, KO=knock-out genotype, min.=minimum, max.=maximum

With respect to sex, the absolute kidney area, relative vessel area, and absolute vessel area in female mice did not differ among the genotypes in females (*n*=5 WT, *n*=7 KO, all p>0.05, Fig. 3A, Supplementary Table 5, Supplementary Figure 4A). When the absolute kidney area was analyzed in male KO kidneys, it was found to be significantly smaller than that of WT kidneys across all frequencies (*n*=8 WT vs. *n*=3 KO MF: 75. 51±9.82 mm^2^ vs. 52.30±18.66 mm^2^, *p*=0.0215). Again, the relative vessel area showed no difference in all frequency bands (all *p* >0.05), while the absolute vascular area of KO kidneys was found to be smaller than that of WT (MF: 54.37±9.35 mm^2^ WT vs. 34.93±13.82 mm^2^ KO, *p*=0.0232) (Fig. 3B, Supplementary Figure 4B and Supplementary Table 6).

**Fig. 4:**
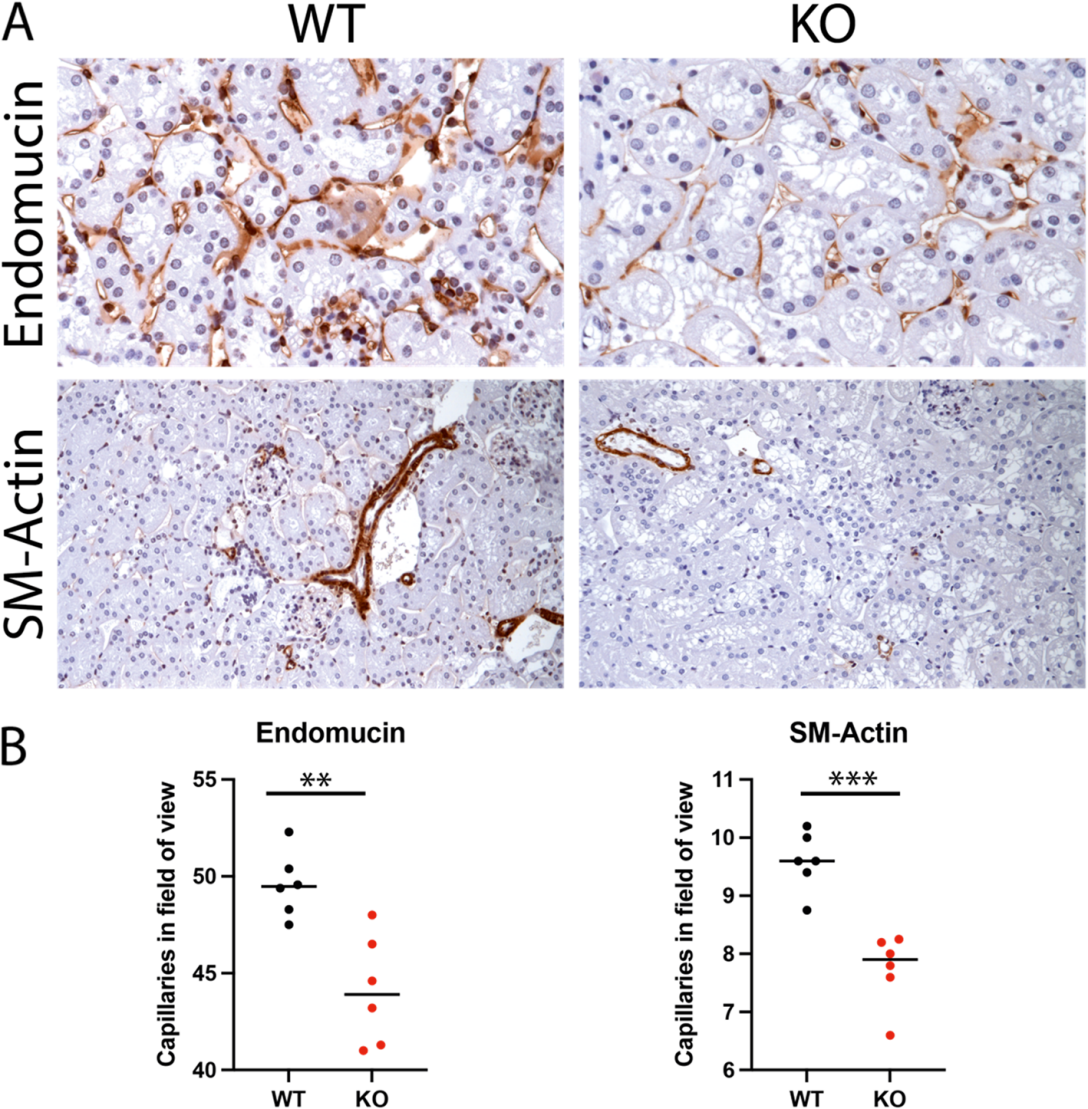
Immunohistochemistry for vascular markers of WT compared to KO mice**. A** Exemplary immunohistochemistry stainings for endomucin (capillaries, brown staining) and smooth muscle actin (SM-Actin, arterioles, brown staining). Left panel indicated WT and right panels KO. **B** Quantification of positive stained vessels in the field of view. Differences were tested with parametric two-sided *t* test. *=*p*<0.05, ***p<0.001, WT=wildtype genotype, KO=knock-out genotype

These findings were comparable to immunohistochemistry for vascular markers (Fig. 4A). Both, endomucin (*p*=0.0021) und small muscle actin (*p*=0.0002) staining revealed decreased vascular density in diseased kidneys (Fig. 4B).

### Multiple wavelength visualization

**Fig. 5:**
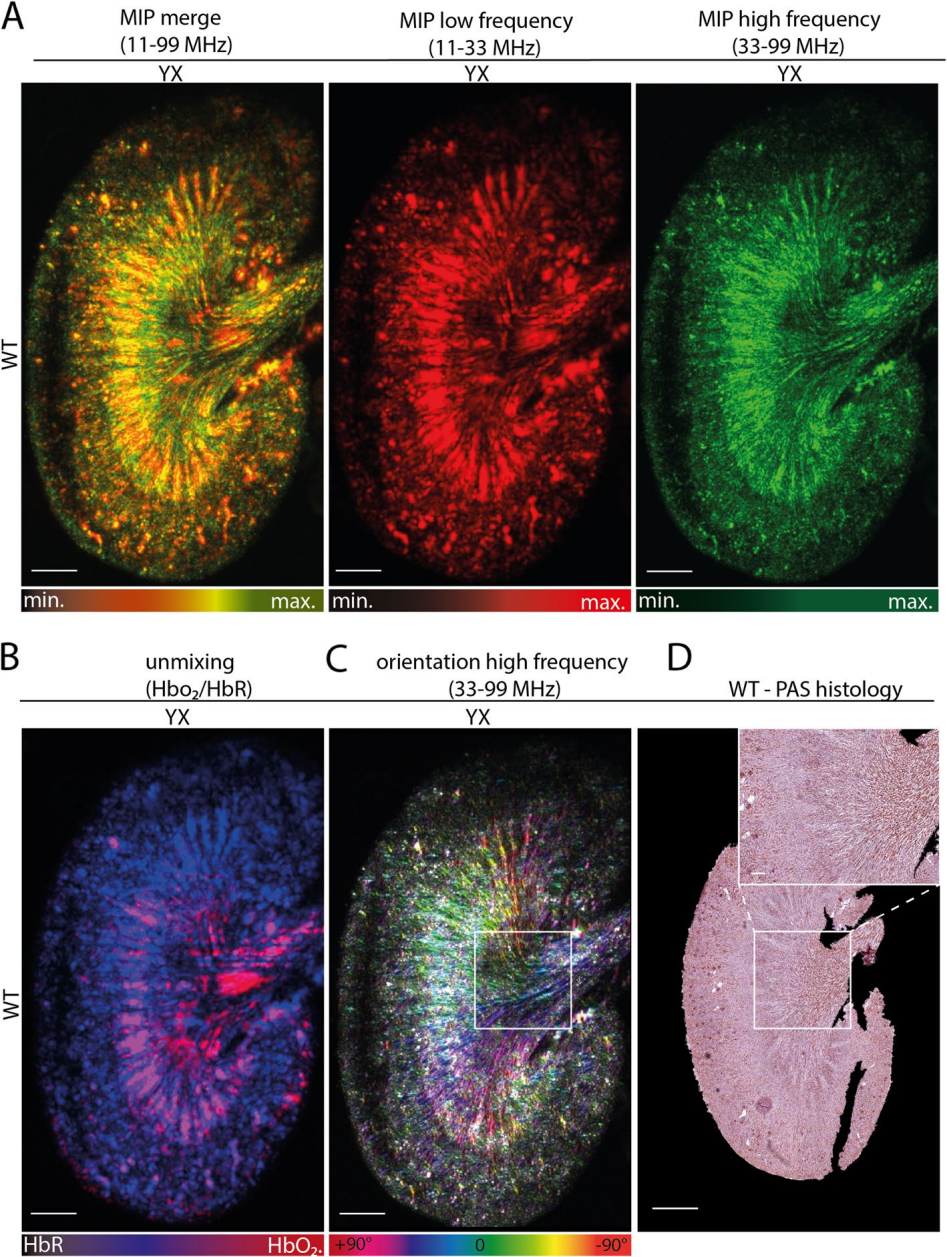
Multispectral raster scanning optoacoustic mesoscopy imaging. **A** Exemplary reconstructed cross-sectional msRSOM maximum intensity projections (MIP) of a wildtype mouse kidney are displayed. Merged frequencies (red-green) combine the information from low frequencies (bigger vessels = red) and higher frequencies (smaller vessels = green) allowing visualization of renal vasculature similar to images from RSOM (Figs. 1 and 3) at the same SWL of 532nm. Scale bars indicate 1mm. **B** Exemplary reconstructed cross-sectional msRSOM maximum intensity projection (MIP) of spectrally unmixed msRSOM parameters (blue=HbR and red=HbO_2_) from four wavelengths (532, 555, 579, 606nm). HbR indicating deoxygenated - venous and HbO_2_ indicating oxygenated–arterial blood in the freshly excised and halved kidney. Scale bars indicate 1mm. **C** The same image as shown in **B** coded for vessel orientation by an image processing software. Orientation of the vessels from the kidney hilus to the periphery is visualized confirming normal anatomical distribution and orientation. Scale bars indicate 1mm. **D** A histological section of the kidney shown in **A**–**C.** PAS staining confirms the anatomical distribution and orientation of kidney tissue and vessel orientation. A 5-fold magnification of a central kidney section (rectangle 2x2mm) is shown. Scale bars indicate 1mm and 0.2mm in the magnification. LF=low frequency, HF=high frequency, MF=merge frequency, HbO_2_=oxygenated hemoglobin, HbR=deoxygenated hemoglobin, WT=wildtype, PAS=period acid-Schiff’s, min.=minimum, max.=maximum

Further, msRSOM was used to elucidate the possibility of imaging oxygenated and deoxygenated vasculature by using multiple wavelengths. Using only one wavelength similar images to RSOM were obtained (Fig. 5A, Supplementary Figure 5). By using an unmixing algorithm for oxygenated and deoxygenated hemoglobin (HbO_2_/HbR) arterial and venous parenchymal regions could be visualized (Fig. 5B). Further analysis of the anatomical vessel orientation can add valuable information to the architecture of the kidney compared to standard histological staining (Fig. 5C, D).

## Discussion

In this ex vivo study (ms) RSOM enabled visualization of vasculature in ex vivo murine kidneys. Reconstructed images allowed detailed mapping of renal vasculature revealing differences between healthy and diseased mice with high resolution.

In KO male mice, the kidneys were smaller and had lower absolute vascularization compared to WT mice. In contrast, the kidneys of female mice showed no differences between genotypes. Our findings suggest a larger renal mass in males with the consecutive higher absolute vascular area. This is in line with findings in a model of simultaneous deficiency of apolipoprotein E and Itga8 wherein renal changes were less pronounced and accompanied by lower expression of inflammatory and fibrotic markers in female mice [35]. Furthermore, this is supported by clinical findings demonstrating sex-specific changes in CKD [36–38]. Using an additional multiwavelength approach, additional biomarkers such as tissue oxygenation levels might be visualized adding further information to conventional imaging modalities. However, the levels of oxygen saturation in the ex vivo imaging data cannot be compared to an in vivo situation. Additionally, the technical limitation of only four wavelengths and the uncertainties of wavelength-depended fluence could produce errors when estimating oxygen saturation [39].

To date, imaging of kidney tissue is mostly performed via ultrasound [40], while imaging techniques such as blood oxygenation level-dependent (BOLD) magnetic resonance tomography still lack significant clinical impact [41, 42]. However, due to its scalability in resolution and imaging depth, OAI provides high potential for further developments to visualize kidney parenchyma [18, 43, 44]. In this regard, contrast-enhanced OAI was capable of quantitatively measuring kidney perfusion potentially suitable for determining acute renal injuries and inflammation [45]. In a translational setting before transplantation, Hysi et al. demonstrated that OAI can be used to determine kidney organ quality [13]. As shown in chronic inflammatory bowel diseases [46, 47], rheumatoid arthritis [48], and muscular diseases [49–51], this could also be achieved via transcutaneous in vivo imaging. In the future, due to the limited penetration depth, it may also be possible to implement this technique in a catheter-based approach to allow contrast-free visualization of single renal vessels [52–54].

The study has several limitations: all experiments were performed on ex vivo kidneys without longitudinal data points. For larger organs, the current field of view of the imaging system is limited and might miss portions of the tissue mass. RSOM is limited by relatively long recording times (approximately 6–7 min per kidney) and therefore requires the implementation of the motion correction methods for in vivo imaging [55].

In conclusion, this study demonstrates the capability of RSOM for label-free visualization of differences in vascular morphology in ex vivo murine renal tissue at high-resolution. Due to its scalability, OAI provides an emerging modality with the potential for further preclinical and clinical imaging applications without the need for ionizing radiation or the application of contrast agents.

## Supplementary Information

**Additional file 1: Supplementary Table 1.** Further information on RSOM Explorer P50 (RSOM P50). Adapted from user manual (RSOM Explorer P50, iThera Medical GmbH, München). **Supplementary Table 2.** Further information on RSOM Explorer ms-P50 (RSOM ms-P50). Adapted from user manual (RSOM Explorer P50, iThera Medical GmbH, München). **Supplementary Table 3.** Sex effects on kidney vascularization, independent of genotype. **Supplement Table 4.** Comparison of the different genotypes, independent of sex. **Supplementary Table 5.** Genotype effect on kidney vascularization for female sex. **Supplementary Table 6.** Genotype effect on kidney vascularization for male sex. **Supplementary Figure 1.** Kidney side and sex distinction, independent of genotype. **Supplementary Figure 2.** Sex effects on kidney vascularization in WT kidneys. **Supplementary Figure 3.** Genotype distinction, independent of sex. **Supplementary Figure 4.** Genotype effect on vascularization for different sexes. **Supplementary Figure 5.** msRSOM.

## Data Availability

All data derived in this manuscript are available from the corresponding author upon reasonable request.

## Abbreviations

BOLD: Blood oxygenation level-dependent
CKD: Chronic kidney disease
GFR: Glomerular filtration rate
HbO_2_/HbR: Oxygenated and deoxygenated
HF: High frequencies
Itga 8 ^-/-^, KO: Integrin subunit alpha 8 deficient, knock-out
Itga 8 ^+/+^, WT: Integrin subunit alpha 8, wildtype
LF: Low frequencies
MF: Merged frequencies
MRI: Magnetic resonance imaging
msRSOM: Multispectral raster scanning optoacoustic mesoscopy
OAI: Optoacoustic imaging
PA/OA: Photoacoustic effect/optoacoustic effect
PAS: Periodic acid-Schiff’s
PBS: Phosphate-buffered saline
ROI: Region of interest
RSOM: Raster scanning optoacoustic mesoscopy
SD: Standard deviation
SM-Actin: Smooth-muscle-actin
