# Supplementary material for: High-resolution label-free mapping of murine kidney vasculature by raster-scanning optoacoustic mesoscopy: an ex vivo study
Source: Mol Cell Pediatr. 2022 Jul 4;9:13. doi: 10.1186/s40348-022-00144-0 (PMC9253231; doi:10.1186/s40348-022-00144-0)
Supplement: Supplementary file 1 — Additional file 1: Supplementary Table 1. Further information on RSOM Explorer P50 (RSOM P50). Adapted from user manual (RSOM Explorer P50, iThera Medical GmbH, München). Supplementary Table 2. Further information on RSOM Explorer ms-P50 (RSOM ms-P50). Adapted from user manual (RSOM Explorer P50, iThera Medical GmbH, München). Supplementary Table 3. Sex effects on kidney vascularization, independent of genotype. Supplement Table 4. Comparison of the different genotypes, independent of sex. Supplementary Table 5. Genotype effect on kidney vascularization for female sex. Supplementary Table 6. Genotype effect on kidney vascularization for male sex. Supplementary Figure 1. Kidney side and sex distinction, independent of genotype. Supplementary Figure 2. Sex effects on kidney vascularization in WT kidneys. Supplementary Figure 3. Genotype distinction, independent of sex. Supplementary Figure 4. Genotype effect on vascularization for different sexes. Supplementary Figure 5. msRSOM. [file 40348_2022_144_MOESM1_ESM.docx]

Supplementary Appendix to **Goebel et al.** “**High resolution label–free mapping of murine kidney vasculature by raster-scanning optoacoustic mesoscopy: an ex vivo study”**

| **Detailed technical information - RSOM Explorer P50** | |
| --- | --- |
| Laser fibers | Two lateral fibers, spot size: 2.5 x 3.9 mm |
| Wavelength | 532 nm, pulses: 2.5 ns, up to 80 µJ/pulse, |
| Repetition rate | Up to 2 kHz |
| Detector  (spherically focused) | LiNb03 detector: center frequency: 50 MHz, bandwidth: 11 – 99 MHz  focal distance: 3mm, active element diameter: 3mm, f-number: 1 |
| Amplifier | 60 dB |
| Image reconstruction | beamforming algorithm |
| Field of view | 12 x 12x 3.0 mm^3^ step size: 20µm |
| Resolution | 10 µm axial, 40 µm lateral |

**Supplementary Table 1:** Further information on RSOM Explorer P50 (RSOM P50). Adapted from user manual (RSOM Explorer P50, iThera Medical GmbH, München).

| **Detailed technical information - RSOM Explorer ms-P50** | |
| --- | --- |
| Laser fiber | One central, spot size: 1 mm |
| Wavelength | 532, 555, 579, 606 nm; 2-5 ns pulse length, max. 1.3 kHz pulse repetition rate; (max. 325 Hz per wavelength), ~ 10-22 µJ pulse energy |
| Repetition rate | Up to 1.3 kHz |
| Detector  (spherically focused, through -hole) | LiNb03 detector: center frequency: 50 MHz, bandwidth: 11-99 MHz  focal distance: 4mm, active element: 4 mm f-number: 1 |
| Amplifier | 30 dB pre- amplifier + 30 dB |
| Image reconstruction | beamforming algorithm |
| Field of view | Up to 12 x 12 mm^3^ step size: 20µm |
| Resolution | 10 µm axial, 40 µm lateral |

**Supplementary Table 2:** Further information on RSOM Explorer ms-P50 (RSOM ms-P50). Adapted from user manual (RSOM Explorer P50, iThera Medical GmbH, München).

| **Absolute kidney area [mm^2^]** | | | |
| --- | --- | --- | --- |
| **Frequency** | **Mean ± SD female** | **Mean ± SD male** | **p Value** |
| **MF** | 52.42 ± 6.24 | 69.18 ± 15.96 | 0.0156 |
| **relative vessel area [%]** | | | |
| **Frequency** | **Mean ± SD female** | **Mean ± SD male** | **p Value** |
| **MF** | 68.18 ± 4.86 | 70.14 ± 4.50 | 0.3277 |
| **LF** | 68.04 ± 3.91 | 68.57 ± 3.67 | 0.7404 |
| **HF** | 66.70 ± 5.55 | 69.05 ± 4.52 | 0.2805 |
| **absolute vessel area [mm^2^]** | | | |
| **Frequency** | **Mean ± SD female** | **Mean ± SD male** | **p Value** |
| **MF** | 35.67 ± 4.22 | 49.07 ± 13.48 | 0.0036 |
| **LF** | 35.72 ± 5.01 | 47.51 ± 11.62 | 0.0042 |
| **HF** | 34.93 ± 4.65 | 48.25 ± 18.18 | 0.0035 |

**Supplementary Table 3: Sex effects on kidney vascularization, independent of genotype**

Detailed data of the measurements on the sex effects on kidney vascularization. Data were tested for normality by the normality and Lognormality test, and if normal, an unpaired *t-test* was performed. A p<0.05 was considered statistically significant. MF= merge frequencies, LF= low frequencies, HF= high frequencies, SD= standard deviation.

| **Absolute kidney area [mm^2^]** | | | |
| --- | --- | --- | --- |
| **Frequency** | **Mean ± SD WT** | **Mean ± SD KO** | **p Value** |
| **MF** | 66.11 ± 14.63 | 53.06 ± 10.84 | 0.0255 |
| **relative vessel area [%]** | | | |
| **Frequency** | **Mean ± SD WT** | **Mean ± SD KO** | **p Value** |
| **MF** | 71.49± 3.92 | 66.04 ± 3.84 | 0.0031 |
| **LF** | 69.63 ± 3.79 | 66.56 ± 2.95 | 0.0464 |
| **HF** | 70.15 ± 4.91 | 64.81 ± 3.74 | 0.0095 |
| **absolute vessel area [mm^2^]** | | | |
| **Frequency** | **Mean ± SD WT** | **Mean ± SD KO** | **p Value** |
| **MF** | 47.45 ± 11.72 | 35.09 ± 7.70 | 0.0089 |
| **LF** | 46.03 ± 10.45 | 35.29 ± 7.17 | 0.0113 |
| **HF** | 46.55 ± 11.65 | 34.48 ± 7.73 | 0.0101 |

**Supplement Table 4: Comparison of the different genotypes, independent of sex**

Detailed result presentation for the described data. Data were tested for normal distribution by the normality and Lognormality test and if a normal distribution was present an unpaired *t-test* was performed and for non-normal distribution, a Mann-Whitney test was applied. Data are mean ±SD. A p<0.05 was considered statistically significant. WT= wildtype genotype, KO= knock-out genotype, SD= standard deviation.

| **Absolute kidney area [mm^2^]** | | | |
| --- | --- | --- | --- |
| **Frequency** | **Mean ± SD WT** | **Mean ± SD KO** | **Adjusted p Value** |
| **MF** | 51.06 ± 3.64 | 53.38 ± 7.74 | 0.5489 |
| **relative vessel area [%]** | | | |
| **Frequency** | **Mean ± SD WT** | **Mean ± SD KO** | **Adjusted p Value** |
| **MF** | 71.26± 4.07 | 65.98 ± 4.33 | 0.0583 |
| **LF** | 70.47 ± 3.68 | 66.30 ± 3.25 | 0.0646 |
| **HF** | 69.90 ± 6.06 | 64.42 ± 4.18 | 0.0918 |
| **absolute vessel area [mm^2^]** | | | |
| **Frequency** | **Mean ± SD WT** | **Mean ± SD KO** | **Adjusted p Value** |
| **MF** | 36.39 ± 3.18 | 35.16 ± 5.02 | 0.6434 |
| **LF** | 36.02 ± 3.61 | 35.50 ± 6.10 | 0.8694 |
| **HF** | 35.62 ± 3.03 | 34.44 ± 5.73 | 0.6863 |

**Supplementary Table 5: Genotype effect on kidney vascularization for female sex**

Detailed result presentation for the described data. These were tested for normal distribution by the normality and Lognormality test and if a normal distribution was present an unpaired *t-test* was performed. Data are mean ±SD. A p<0.05 was considered statistically significant. WT= wildtype genotype, KO=knock-out genotype, SD= standard deviation.

| **Absolute kidney area [mm^2^]** | | | |
| --- | --- | --- | --- |
| **Frequency** | **Mean ± SD WT** | **Mean ± SD KO** | **p Value** |
| **MF** | 75.51 ± 9.82 | 52.30 ± 18.66 | 0.0215 |
| **relative vessel area [%]** | | | |
| **Frequency** | **Mean ± SD WT** | **Mean ± SD KO** | **p Value** |
| **MF** | 71.63± 4.10 | 66.18 ± 3.17 | 0.0698 |
| **LF** | 69.11 ± 4.00 | 67.14 ± 2.62 | 0.4578 |
| **HF** | 70.30 ± 4.50 | 65.72 ± 2.92 | 0.1416 |
| **absolute vessel area [mm^2^]** | | | |
| **Frequency** | **Mean ± SD WT** | **Mean ± SD KO** | **p Value** |
| **MF** | 54.37 ± 9.35 | 34.93 ± 13.82 | 0.0232 |
| **LF** | 52.29 ± 7.97 | 34.79 ± 10.92 | 0.0158 |
| **HF** | 53.39 ± 9.43 | 35.55 ± 13.04 | 0.0248 |

**Supplementary Table 6: Genotype effect on kidney vascularization for male sex**

Detailed result presentation for the described data. These were tested for normal distribution by the normality and Lognormality test and if a normal distribution was present an unpaired *t-test* was performed. Data are mean ±SD. A p<0.05 was considered statistically significant. WT= wildtype genotype , KO= knock-out genotype, SD= standard deviation.

**
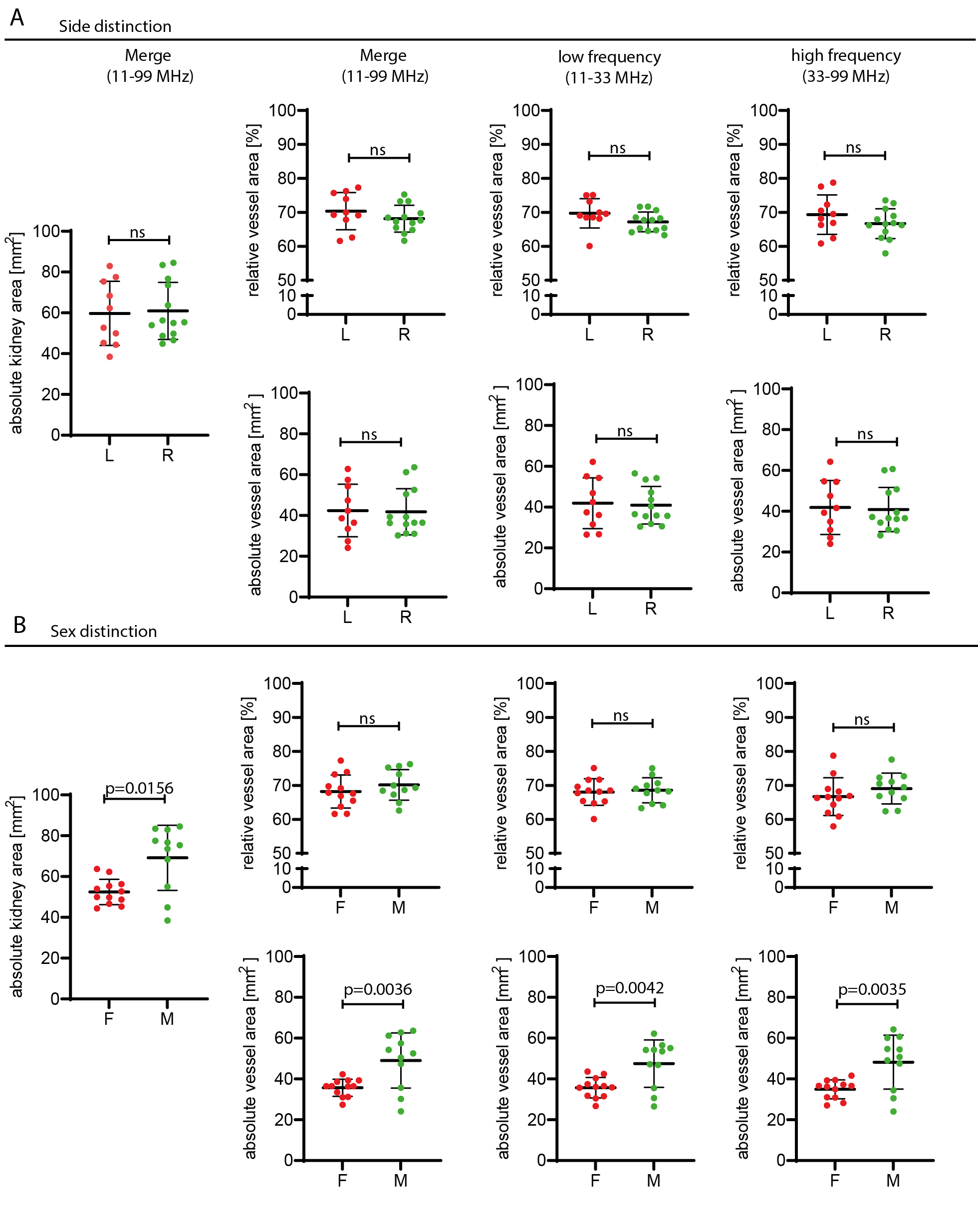
**

**Supplementary Figure 1: Kidney side and sex distinction, independent of genotype**

**(A)** Comparisons of each kidney side after measurement of absolute kidney area, relative vessel area, and absolute vessel area shown graphically.

**(B)** Sex differences in measurements are shown. The measurement was performed using the merge frequency band, LF and HF. Data were tested for normality by the normality and Lognormality test, and if normal, an unpaired *t-test* was performed and for non-normal distribution, a Mann-Whitney test was applied. Data are mean ±SD. P-values for significant comparisons are shown. A p<0.05 was considered statistically significant. NS= not significant, LF= low frequencies, HF= high frequencies, F= female, M= male, L= Left, R= right

**
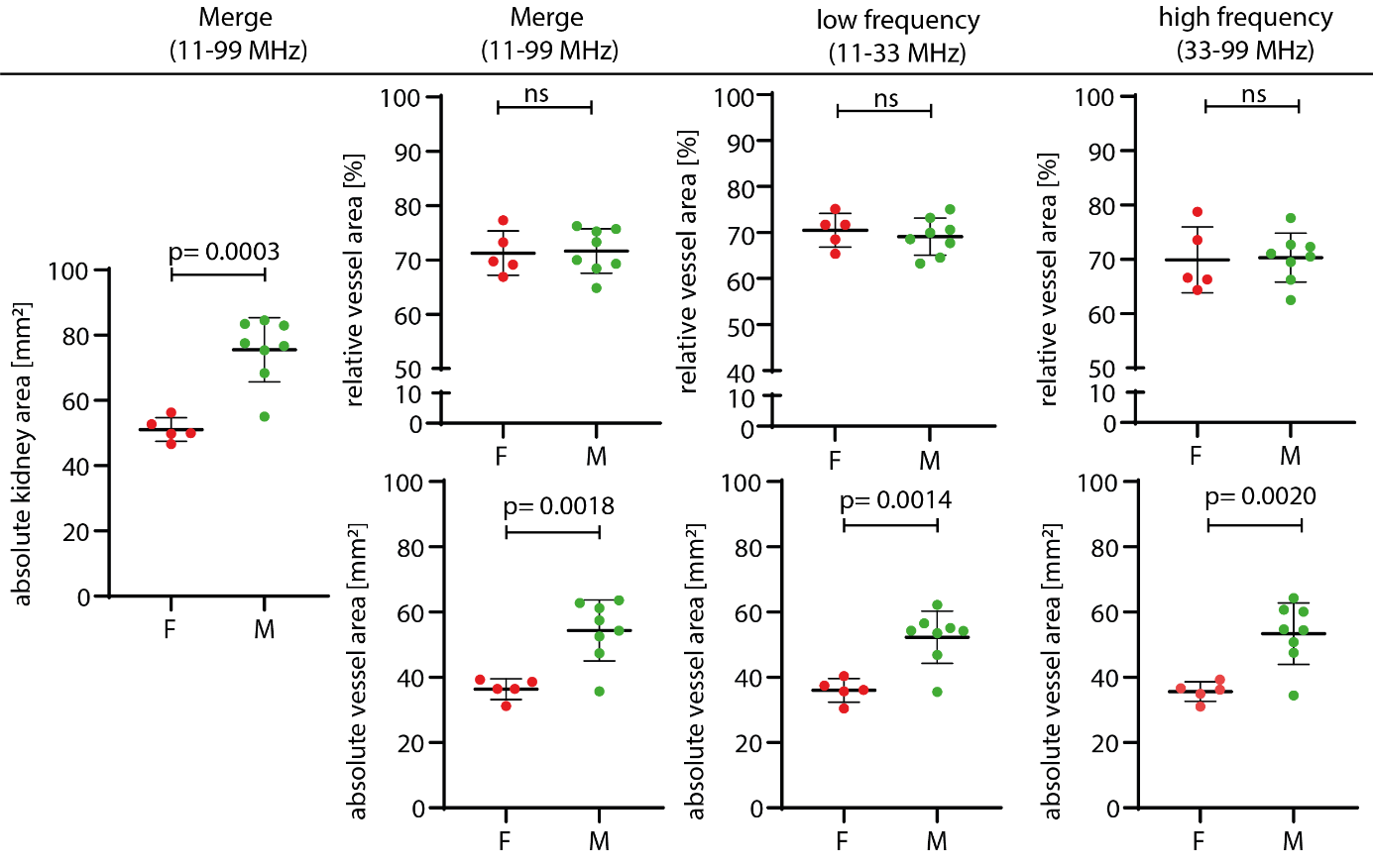
**

**Supplementary Figure 2: Sex effects on kidney vascularization in WT kidneys**

Measurements on the effects of sex on absolute kidney area, relative vessel area, and absolute vessel area in WT kidneys. The measurement was performed using the merge frequency band, LF and HF. Data were tested for normality by the normality and Lognormality test, and if normal, an unpaired *t-test* was performed. Data are mean ±SD. A p<0.05 was considered statistically significant. NS= not significant, LF= low frequencies, HF= high frequencies, WT= wildtype genotype

**
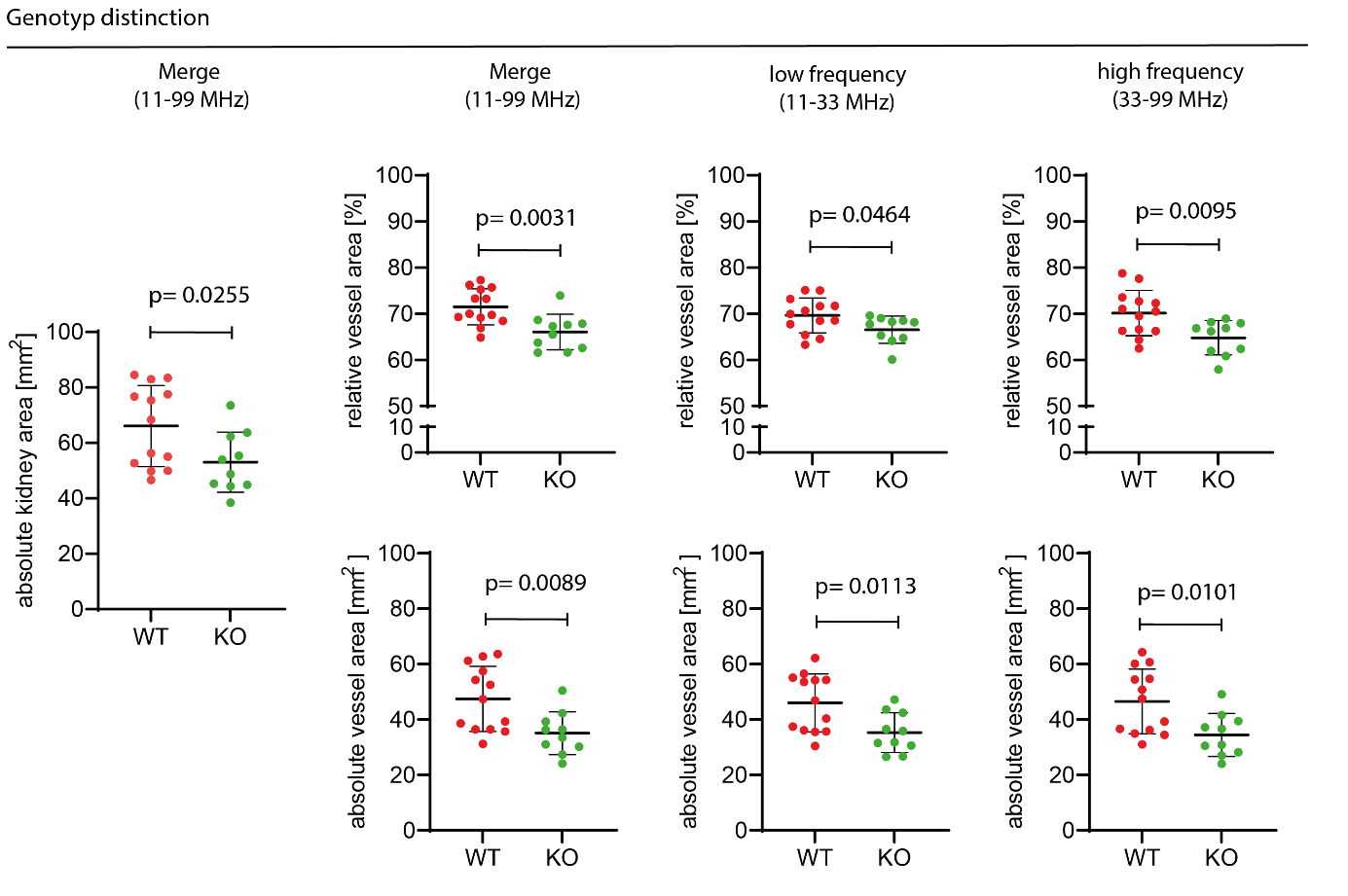
**

**Supplementary Figure 3: Genotype distinction, independent of sex**

The measurement of absolute kidney area, relative vessel area and absolute vessel area is shown here graphically based on the different genotypes in MF, LF and HF. Data were tested for normality by the normality and Lognormality test, and if normal, an unpaired *t-test* was performed and for non-normal distribution, a Mann-Whitney test was applied. Data are mean ±SD. A p<0.05 was considered statistically significant. NS= not significant MF= merge frequencies, LF= low frequencies, HF= high frequencies. WT= wildtype genotype, KO= knock-out genotype.

**
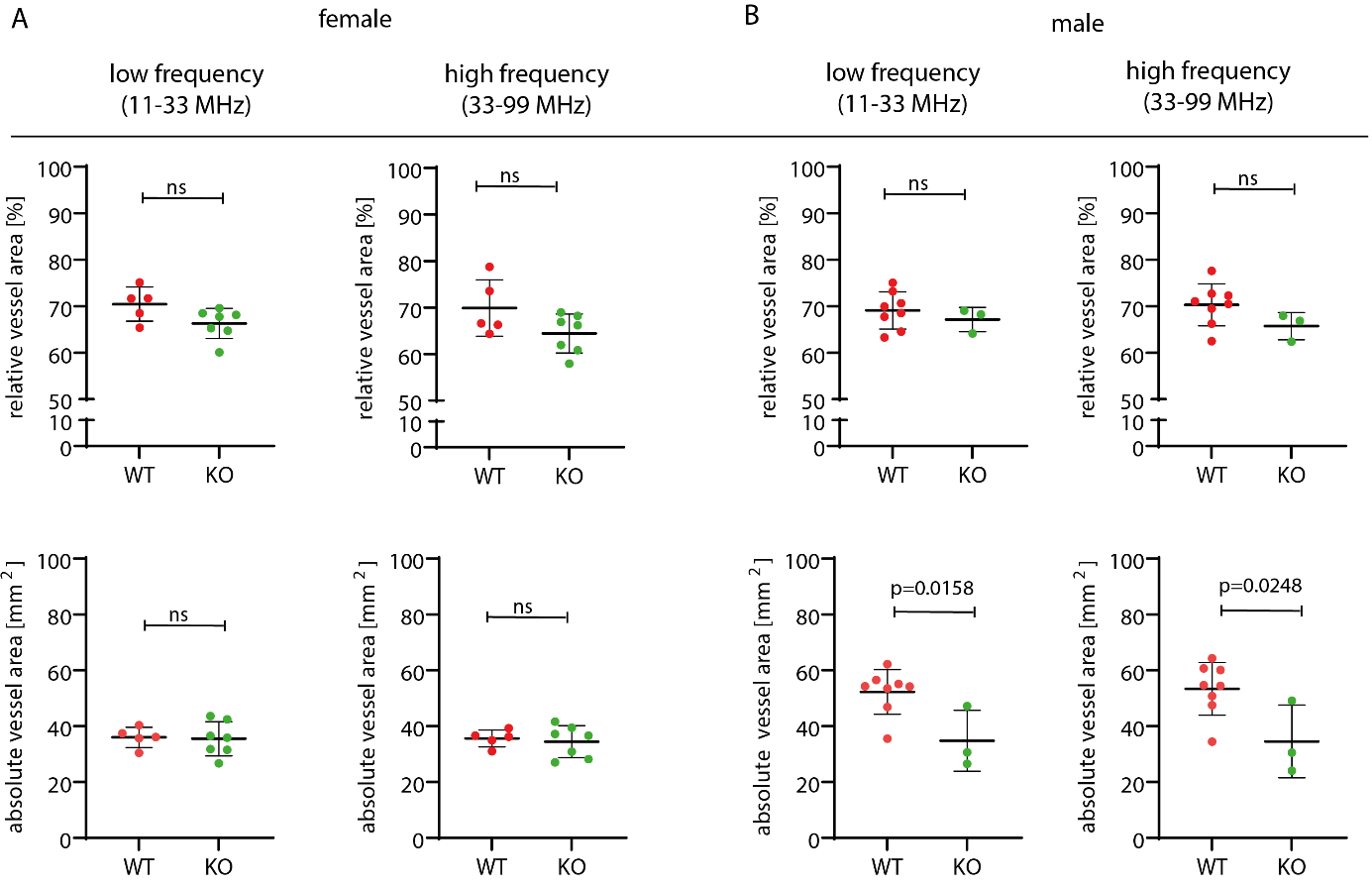
**

**Supplementary Figure 4: Genotype effect on vascularization for different sexes**

**(A)** Measurements of the different genoytpes for the female kidneys, based on LF, HF. **(B)** The same measurements were performed for the male kidneys. Data were tested for normal distribution by the normality and Lognormality test and an unpaired *t-test* was performed. Data are mean ±SD. A p<0.05 was considered statistically significant. LF= low frequencies, HF= high frequencies.


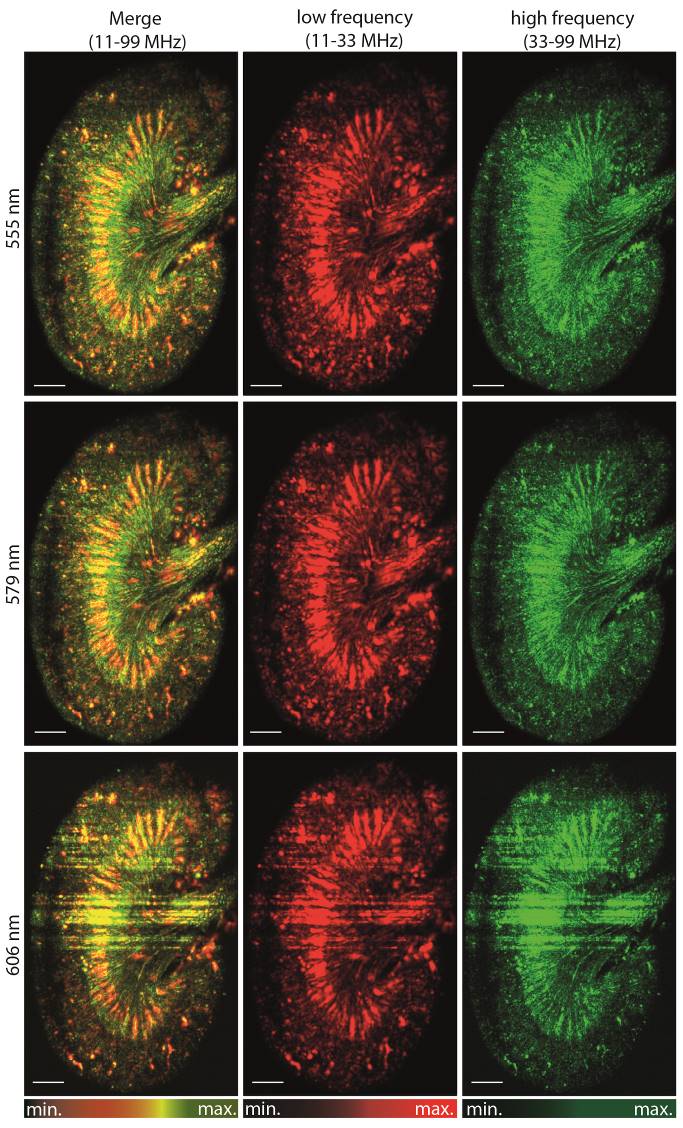


**Supplementary Figure 5: msRSOM**

Additional wavelengths (555nm, 579nm, 606nm) as imaged with msRSOM system.

Min.=minimum, max.=maximum.
